# The dynamic interplay between mental health difficulties and the family environment in early adolescence

**DOI:** 10.1002/jcv2.70037

**Published:** 2025-08-12

**Authors:** Ludvig Daae Bjørndal, Omid V. Ebrahimi, Sarah Bauermeister

**Affiliations:** ^1^ Department of Psychology PROMENTA Research Center University of Oslo Oslo Norway; ^2^ Psychiatric Genetic Epidemiology Group Research Department Lovisenberg Diaconal Hospital Oslo Norway; ^3^ Department of Psychiatry Dementias Platform UK University of Oxford Oxford UK; ^4^ Department of Experimental Psychology University of Oxford Oxford UK

**Keywords:** adolescent mental health, family environment, graphical vector autoregression, network analysis

## Abstract

**Background:**

Adolescents experiencing mental health problems have an elevated risk of persisting difficulties as they transition into adulthood, stressing the importance of identifying modifiable factors impacting mental health during adolescence. The family environment is recognised as a key influence on adolescent mental health in theory and interventions. Notably, few studies have disentangled within‐person and between‐person effects in relating adolescent mental health and the family environment.

**Methods:**

We analysed data from 1067 adolescents across three waves using panel graphical vector autoregressive modelling, separating contemporaneous and temporal within‐person and between‐person associations in relationships between mental health difficulties (i.e., emotional, hyperactivity/inattention, conduct problems) and family‐related factors (i.e., aspects of the general family environment, parent‐child relationships, and sibling dynamics). We also assessed how the different mental health difficulties and family environment factors were themselves interrelated over time. The mean age (in years) was 10.51 at Wave 1, 12.49 at Wave 2, and 14.49 at Wave 3.

**Results:**

Emotional symptoms predicted increases in hyperactivity/inattention and more sibling problems over time. Lack of family support and negative feelings towards family were reciprocally related, indicative of a reinforcing loop. Both mental health difficulties and family environment factors exhibited considerable stability. In contemporaneous within‐person associations, mental health difficulties were strongly interrelated, as were aspects of the family environment. Furthermore, conduct problems were linked to externalising behaviours (e.g., fighting with parents, bothering siblings) and emotional symptoms to internalising experiences of family dynamics (e.g., feeling negative towards family, being bothered by siblings). Negative feelings towards family and hyperactivity/inattention were strongly predicted by included variables, while emotional symptoms, fighting with parents, and lacking family support were predictive of other variables.

**Conclusions:**

Our findings point to the importance of emotional problems in adolescence, which may contribute to worsened hyperactivity/inattention and more problems with siblings over time, and the interrelatedness of mental health and the family environment. Alleviating internalising problems in affected adolescents may help mitigate development of other mental health difficulties and negative sibling dynamics.

## Key Points

**What's known**

- The family environment is recognised as a key influence on adolescent mental health in theory and interventions, yet there is scarce longitudinal evidence pertaining to how family dynamics influence mental health in this period.

**What's new**

- In analyses of data from 1067 adolescents across three waves, we found that emotional problems in adolescence predict increases in hyperactivity/inattention and more problems with siblings over time. Our findings underscore the interrelatedness of mental health difficulties and the family environment, as well as temporal effects within and across symptom domains and family environment factors.

**What's relevant**

- Alleviating internalising problems in affected adolescents may help mitigate development of other mental health difficulties and negative sibling dynamics. Future studies could examine within‐person associations between adolescent mental health and the family environment across timescales and in clinical samples.

## Graphical abstract

This paper unravels relationships between adolescent mental health and family environment factors. We find that emotional problems in adolescence predict both hyperactivity/inattention and sibling problems over time.

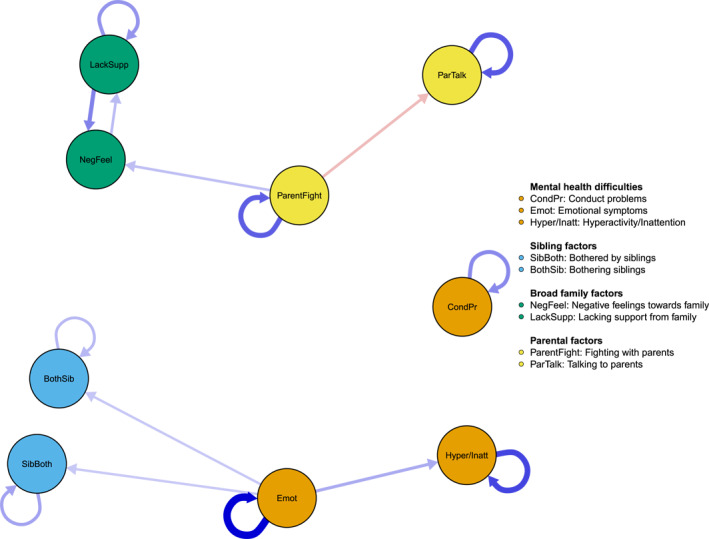

## INTRODUCTION

Adolescence, commonly defined as the time between puberty and adulthood, is a crucial period of biological, psychological, and social change (Blakemore, 2019). It is during this time that the foundations for mental health across the lifespan are established (Beckwith et al., 2024; Sawyer et al., 2012). The median age of onset for a range of mental disorders is before 20 years (Kessler et al., 2007; Solmi et al., 2022). More than 1 in 10 children and adolescents have a diagnosable mental disorder (Kieling et al., 2024). Crucially, around half of boys and a majority of girls with a mental disorder in adolescence experience at least one mental health episode as young adults (Patton et al., 2014), indicating persisting mental health difficulties for many adolescents transitioning into adulthood.

Comorbidity of mental health problems in this life stage is widespread. For instance, depression in adolescents frequently co‐occurs with anxiety, attention‐deficit hyperactivity disorder (ADHD), and conduct disorder (Avenevoli et al., 2015; Smalley et al., 2007). Moreover, different mental health problems can potentially co‐develop and exacerbate each other over time. For instance, Speyer et al. (2021) identified reciprocal relationships between internalising and ADHD symptomatology over time in childhood. Widespread comorbidity and symptom overlap across diagnoses underline the importance of broadly assessing the development of mental health difficulties in adolescence.

Adolescents are intertwined in a complex web of social influences: from family to peers to the wider social context (Viner et al., 2012). A negative family environment constitutes one risk factor for poor adolescent mental health (Kieling et al., 2011). This is supported by transactional models which highlight reciprocal relationships (involving child‐to‐parent and parent‐to‐child effects) between children and parents with respect to developmental outcomes in children (Sameroff & Mackenzie, 2003). Empirically, the notion of reciprocal effects between children and caregivers has recently been supported by a small number of longitudinal (e.g., Speyer, Hall, et al., 2022; Speyer, Hang, et al., 2022; Veenman et al., 2024) and genetically informed studies (e.g., Bjørndal et al., 2024; Eilertsen et al., 2022).

Several aspects of the family environment may contribute to such transactional effects. For instance, it has been shown that harsh parenting practices and physical discipline are related to mental health difficulties in childhood and adolescence over time and that these associations may be bidirectional (i.e., parenting and adolescent mental health reciprocally predicting each other; Hipwell et al., 2008; Lansford et al., 2011; Speyer, Hang, et al., 2022; Wang & Kenny, 2014), caring parenting styles are linked with lower risk of mental disorders in adolescence (Eun et al., 2018), and more parental support is associated with less adolescent‐reported depressive symptoms (Boele et al., 2023). Poorer adolescent‐assessed communication with parents has been associated with mental health difficulties (Zapf et al., 2024). The importance of the family environment as a crucial context for development of child and adolescent mental health is emphasised in models based on systemic theory (Bronfenbrenner, 1986; Restifo & Bögels, 2009). Several preventative and treatment efforts for mental health difficulties in children target aspects of the family context, such as the Triple P‐Positive Parenting Program (Sanders et al., 2014) and systemic therapy (Riedinger et al., 2017).

The family environment evolves throughout childhood and adolescence as both children and adults age and transition through different life stages. Consequently, the dynamics within the family may shift over time. For instance, a significant number of families experience parental divorce or separation before adolescence concludes (Gottman & Levenson, 2000), which can be preceded by negative parental interactions. Recent studies highlight how factors like parental mental health (Speyer, Hall, et al., 2022) and parenting practices (Speyer, Hang, et al., 2022) exhibit both stability and change throughout childhood and adolescence.

The preponderance of existing studies of adolescent mental health and the family environment exhibits four key limitations. First, many studies have been conducted based on cross‐sectional data, which cannot be used to distinguish between‐person and within‐person effects, which may sometimes yield conflicting results (Curran & Bauer, 2011; Hamaker et al., 2015; Kievit et al., 2013). For instance, although adolescents who experience more family support may tend to report better mental health compared to their peers (i.e., a between‐person relationship), an adolescent might experience increased emotional distress during periods of unusually high family involvement (e.g., overprotectiveness) compared to their typical family interactions (i.e., a within‐person relationship). Furthermore, conflating within‐ and between‐person effects means that stable between‐family differences (e.g., in genetic vulnerabilities) can confound estimates of within‐family associations (Speyer, Hang, et al., 2022). Second, studies have typically examined associations between mental health and single aspects of the family environment, overlooking the complexity of the family system. Specifically, while several studies have examined associations between parenting practices and adolescent mental health over time (e.g., Hipwell et al., 2008; Lansford et al., 2011; Speyer, Hang, et al., 2022; Wang & Kenny, 2014), fewer studies have examined longitudinal associations between mental health and multiple family factors, including broader aspects of the family (e.g., perceived family support) and sibling relationships. Third, there is a scarcity of studies incorporating mental health assessments that extend beyond a single diagnostic category.

An innovative methodological approach which helps overcome several of these limitations was recently proposed: the panel graphical vector autoregressive (panelGVAR) model (Epskamp, 2020; Epskamp, Waldorp, et al., 2018). This is a longitudinal network approach which allows for separating within‐ from between‐person effects, and the modelling of associations between mental health problems (e.g., Skjerdingstad et al., 2024; Speyer et al., 2021), and related variables (e.g., Freichel, Skjerdingstad, et al., 2023, Freichel, Veer, et al., 2023) across time. The panelGVAR model is particularly useful for assessing interrelationships between multiple variables and how these interrelate over time. This is also in accordance with a systems‐based approach, emphasising the interaction between multiple components of mental health and risk factors over time (Borsboom et al., 2022).

In this exploratory study, we explore the interplay between mental health difficulties and various aspects of the family environment, including broader family factors (i.e., experienced family support and feelings about family), parental dynamics (i.e., conflict and communication with parents), and sibling relationships (i.e., being bothered by or bothering siblings). Using data from 1067 participants measured across early adolescence, we unravel temporal and contemporaneous within‐person relationships between mental health and the family environment. Furthermore, we examine associations between different mental health domains and delineate how family environment factors are interrelated over time in adolescence.

## MATERIALS AND METHODS

### Sample

We used data from the UK Household Longitudinal Study (UKHLS). UKHLS is an annual longitudinal study, which initiated with the recruitment of around 40,000 households across the UK in 2009 (University of Essex, 2023). Data are collected annually with each data collection wave lasting for up to 2 years. Youth surveys are completed by adolescents aged from approximately 10–15 years.

The UKHLS youth surveys collect data on mental health difficulties and perceptions of the family environment biannually. Data were combined from two adolescent subsamples, each with responses at three consecutive waves of data collection which involved assessments of mental health and perceptions of the family environment (a flow chart for the sample selection process is depicted in Figure S1). The first subsample, consisting of 618 adolescents, provided data during waves 1 (2009), 3 (2011), and 5 (2013). The second independent subsample, comprising 449 adolescents, contributed data during waves 7 (2015), 9 (2017), and 11 (2019). The full sample therefore comprised children followed sequentially from a first wave of data collection (W1) through a second (W2) and third wave (W3). Participants aged out of eligibility for the youth questionnaire by the fourth wave of data collection. In the case of multiple children from the same household, we randomly selected one child at W1 (i.e., each household had only one child included in the study).

At W1, 52% (*N* = 558) of the sample was female. The mean age of the participants at W1 was 10.51 (SD = 0.53), the mean age at W2 was 12.49 (SD = 0.56), and the mean age at W3 was 14.49 (SD = 0.55).

### Measures

A full list of items and their corresponding response formats can be found in Table S1. We provide descriptive statistics for all variables in Table S2. We report correlations for each variable across waves of data collection in Table S3.

#### Mental health difficulties

We used three subscales from the Strengths and Difficulties Questionnaire (SDQ) to assess mental health (Goodman, 1997, 2001; Stone et al., 2015). Each subscale consisted of five items. Composite scores assessing emotional symptomatology (i.e., somatic complaints, excessive worrying, feelings of unhappiness, nervousness in new situations, loss of confidence, and fears or being easily scared), conduct problems (i.e., angry, usually do as I am told, fight a lot, accused of lying, take things), and hyperactivity (i.e., restless, fidgeting, distracted, thinks things out, see tasks through) over the last 6 months were included. These were calculated for each adolescent when at least three out of five items were completed. All SDQ items measured mental health difficulties experienced over the past 6 months, with response alternatives ranging from ‘Not True’ (0) to ‘Certainly True’ (2). Cronbach's alpha estimated based on polychoric correlation matrices ranged from 0.76 to 0.83 across data collection waves for the emotional symptomatology subscale, 0.77–0.79 for the conduct problems subscale, and 0.76–0.82 for the hyperactivity subscale.

#### Family environment factors

We also included adolescent‐reported assessments of various aspects of the family environment. This encompassed questions on the extent to which respondents feel supported by their family (single item), with response options ranging from ‘In most or all of the things I do’ (0) to ‘I do not feel supported by my family’ (2), and how respondents feel about their family overall, with options ranging from ‘Completely happy’ (1) to ‘Not at all happy’ (7). Four items assessed experiences of sibling conflict (e.g., being hit, kicked, or pushed; having belongings taken; being called nasty names; being made fun of), which were combined into a sum score. A similar set of four items assessed how often respondents engaged in these behaviours towards their siblings, also combined into a sum score. The response format for these items ranged from ‘Never’ (1) to ‘A lot (a few times every week)’ (4). Additionally, we included two items assessing how often adolescents talked to their mother and father about important matters, combined into a summated score, as well as two items assessing how often they quarrelled with their mother and father, also combined into a sum score. The response format for these latter four items ranged from ‘Hardly ever’ (1) to ‘Most days’ (4). Composite scores were calculated when at least half of items were present.

Cronbach's alpha estimated based on polychoric correlation matrices ranged from 0.83 to 0.84 across data collection waves for the being bothered by siblings sum score, 0.83–0.84 for the bothering siblings sum score, 0.81–0.85 for the talking to parents sum score, and 0.77–0.85 for the quarrelling with parents sum score.

### Statistical analysis

#### PanelGVAR

We assessed temporal and contemporaneous within‐person associations using a panelGVAR model (Epskamp, 2020; Epskamp, van Borkulo, et al., 2018; Epskamp, Waldorp, et al., 2018). PanelGVAR is a model which incorporates random effects on the mean structure. It is structurally similar to the random‐intercept cross‐lagged panel model, but models within‐ and between‐person covariance structures as Gaussian graphical models (Epskamp, 2020; Freichel, Veer, et al., 2023). In a panelGVAR model, all variables are predicted by themselves and other variables at the previous time‐point capturing autoregressive and cross‐lagged effects, respectively. The model can be estimated when measurements are available for at least three waves of data collection. In this study, the average time lag between measurements was 2 years. We incorporated sum scores for mental health subscales to assess broad patterns of associations with family environment factors and as it would not be computationally feasible to assess symptom‐level associations.

The panelGVAR model estimates three distinct networks (Epskamp, 2020). It estimates both a temporal and a contemporaneous within‐person network. The former reflects how deviations from an individual's mean on a variable at one timepoint predicts deviations at a subsequent timepoint. This network also includes autoregressive effects, indicating whether a variable predicts itself at a future timepoint. The contemporaneous network captures state‐like associations occurring within the same measurement period. For instance, if assessing the relationship between family support and sadness, within‐person effects could highlight that when receiving less family support than usual, this is associated with feeling greater sadness compared to one's usual sadness level (contemporaneous effect), and/or predicts more sadness at the next time point (temporal effect). Finally, the model estimates a between‐person network, which reflects mean‐level associations across individuals and may encompass broader and stable trait‐like effects (Freichel, Veer, et al., 2023). The resulting networks represent conditional associations, that is, unique relationships between variables, after controlling for all other variables included in the network model.

The panelGVAR model assumes stationarity, that is, that the means and variances do not change across timepoints (Epskamp, 2020). To facilitate this, we removed any linear and quadratic effects of time by regressing each included variable on data collection timepoint (i.e., regressing on timepoint and its squared term), following procedures conducted in previous studies applying panelGVAR (e.g., Freichel, Skjerdingstad, et al., 2023; Hoffart et al., 2024). All variables were standardised separately across all measurement timepoints.

We first estimated an unconstrained saturated panelGVAR model (i.e., including all possible relationships). To reduce the risk of false positives and increase sparsity in the estimated networks, we subsequently estimated a pruned network model followed by step‐up model search estimation (with α = .05), following common procedures in the network literature (Blanken et al., 2022). The focus of this paper is on within‐person effects and the between‐person variance‐covariance structure was modelled using a Cholesky decomposition (we note that this should not be substantially interpreted when estimated with a Cholesky decomposition; Freichel & Epskamp, 2024). We used full information maximum likelihood for model estimation of panelGVAR, which is a state‐of‐the‐art approach to handle missing data, and reduces bias compared with complete case analysis (Baraldi & Enders, 2010).

Multiple fit indices were inspected to determine the fit of the model (Du et al., 2024), including the root‐mean‐square error of approximation (RMSEA), the comparative fit index (CFI), and the Tucker‐Lewis Index (TLI), using conventional thresholds: CFI > 0.95, TLI > 0.95, and RMSEA < 0.06 (Hu & Bentler, 1999). The panelGVAR model was estimated using the *psychonetrics* package (Epskamp, 2024) in the R Statistical Environment (version 4.4.1; R Core Team, 2023). Networks were visualised using the *qgraph* package (Epskamp et al., 2012). This study was not pre‐registered.

#### Centrality estimates

We obtained several centrality metrics to examine the connectedness of each node (Ebrahimi et al., 2021). We estimated out‐strength (i.e., the sum of the absolute values of all outgoing edges from a node) and in‐strength (i.e., the sum of the absolute values of all incoming edges to a node) for the temporal network, excluding autoregressive effects. We estimated strength centrality (i.e., the sum of the absolute values of all edges connected to a node) for the contemporaneous network.

## RESULTS

The saturated panelGVAR model exhibited acceptable fit indicated by TLI and good fit indicated by CFI and RMSEA (TLI = 0.90, CFI = 0.94, RMSEA = 0.042). The pruned model displayed improved fit compared with the saturated model (TLI = 0.92, CFI = 0.93, RMSEA = 0.037). The mean age (in years) was 10.51 at Wave 1, 12.49 at Wave 2, and 14.49 at Wave 3.

### Contemporaneous associations within the same time window

**FIGURE 1:**
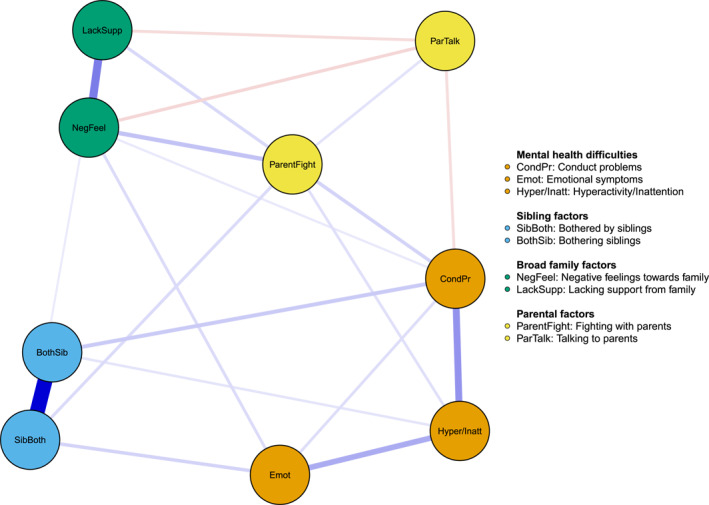
Pruned contemporaneous network: associations within the same time window. The colours (red = negative, blue = positive) indicate the direction of associations, while the thickness of edges indicate the magnitude. Edge weights ranged from −0.11 to 0.67.

The contemporaneous network captures how the included variables are interrelated within the same time window (see Figure 1). There were substantial associations between mental health difficulties, that is, emotional symptoms, conduct problems, and hyperactivity/inattention, highlighting their co‐amplification.

Conduct problems were related to several family environment factors, including bothering siblings, fighting with parents, experiencing lack of support, less talking to parents, and negative feelings towards family. Hyperactivity/inattention was linked to more fighting with parents and bothering siblings. Emotional symptoms were associated with negative feelings about one's family and being bothered by siblings.

Among family environment factors, feeling negatively about one's family was associated with less family support, more fighting with parents, and less talking with parents. Being bothered by and bothering siblings were strongly interrelated, with the former being associated also with more fighting with parents and less family support. Talking to parents more was additionally associated with more family support, as well as more fighting with parents.

### Temporal associations across time

**FIGURE 2:**
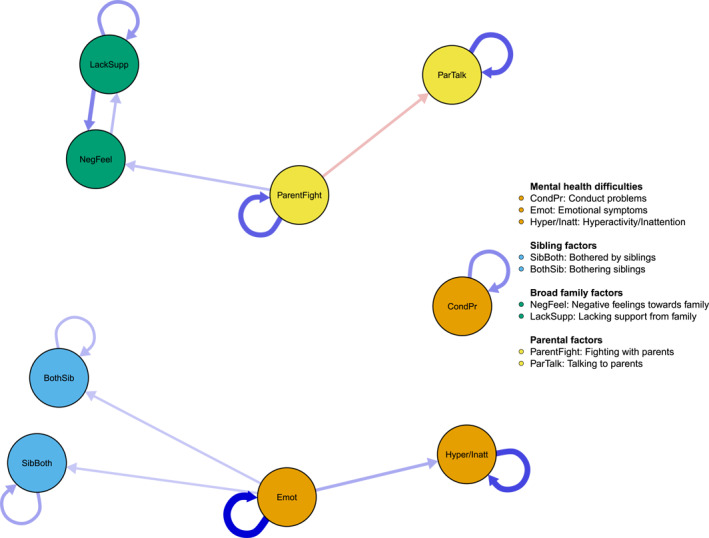
Pruned temporal network: directed associations across time. The colours (red = negative, blue = positive) indicate the direction of associations, while the thickness of edges indicate the magnitude. Edge weights ranged from −0.08 to 0.29.

The temporal network visualises how variables included in the network are predicted by themselves or other variables at the preceding timepoint (see Figure 2). Among mental health difficulties, emotional symptoms emerged as a predictor of hyperactivity/inattention at the next timepoint. All mental health problems (i.e., emotional symptoms, conduct problems, and hyperactivity/inattention) exhibited strong autoregressive effects, highlighting how these maintain themselves over time.

Emotional problems were directly linked to being bothered by siblings and bothering siblings, suggesting that adolescents who experience higher levels of emotional symptoms tend to feel more bothered by and bother siblings more at the next timepoint.

Our results also provide insights into family dynamics over time. A bidirectional loop was observed between lack of support and negative feelings towards family, such that these reciprocally predicted increases in each other at the next timepoint (i.e., indicative of a reinforcing loop). More fighting with parents predicted more negative feelings towards family and less talking to parents. Except for negative feelings towards family, all family‐related factors showed substantial autoregressive effects, indicative of their stability across time.

### Centrality estimates

**FIGURE 3:**
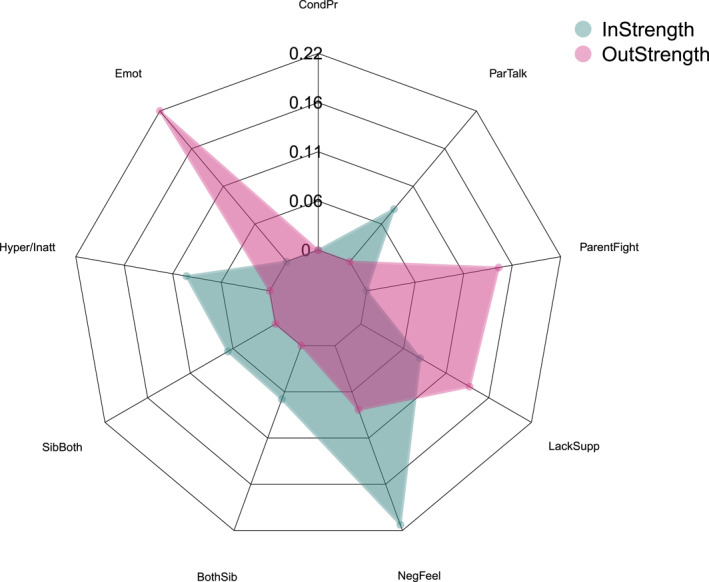
Pruned temporal network: out‐ and in‐strength estimates. BothSib, bothering siblings; CondPr, conduct problems; Emot, emotional symptoms; Hyper/Inatt, hyperactivity/inattention; LackSupp, lacking support from family; NegFeel, feeling negative towards family; ParentFight, fights or quarrels with parents; ParTalk, talking to parents about things that matter to me; SibBoth, being bothered by siblings.

Out‐ (outgoing) and in‐strength (incoming) centrality estimates for the temporal network are visualised in Figure 3. Emotional symptoms, fighting with parents, and lacking family support exhibited the strongest outwards influence in the network. Negative feelings towards family, hyperactivity/inattention, and talking to parents were most strongly influenced by other variables included in the network model (i.e., highest in‐strength). Centrality estimates for the contemporaneous network are reported in Figure S2.

## DISCUSSION

We investigated the relationship between mental health and family environment factors in a sample of 1067 adolescents measured from early to mid‐adolescence. Unravelling how mental health difficulties evolve within individuals and interact with family dynamics is of great importance for understanding the development and persistence of mental health problems during this life stage.

Our findings highlight the interrelatedness of mental health difficulties and their concurrent interrelationships. We observed strong associations between emotional symptoms, conduct problems, and hyperactivity/inattention in the contemporaneous network, indicative of their co‐amplification within the same time window (i.e., 2 years). There is high comorbidity among emotional disorders, ADHD, and conduct disorders in adolescence (Avenevoli et al., 2015; Smalley et al., 2007). From a network perspective, a central mechanism underlying this comorbidity may be the interactions between individual symptoms across different domains (Borsboom, 2017; Cramer et al., 2010).

Notably, we also found that emotional symptoms predicted hyperactivity/inattention at the next timepoint, suggesting that emotional symptomatology may contribute to worsened hyperactivity/inattention over time early adolescence. However, these symptom relationships may also be more complex. For instance, previous studies have noted reciprocal relationships between internalising and ADHD symptomatology (Murray et al., 2022; Speyer et al., 2021). Of note, several intervention studies have found that psychological treatments primarily targeting anxiety symptoms (e.g., cognitive behavioural therapy) may also reduce ADHD symptoms in children and adolescents (Gould et al., 2018; Sprich et al., 2016).

We found that children who experienced mental health problems in one symptom domain (i.e., emotional, conduct problems, and/or hyperactivity), tended to experience more problems in the same domain 2 years later (i.e., autoregressive effects). In sample of adolescents followed for 14 years, Patton et al. (2014) reported that rates of persisting or recurrent clinically assessed disorders were 37% for girls and 14% for boys, respectively. The stability of mental health difficulties (both with respect to self‐reported symptom measures and clinical diagnoses) for a substantial proportion of adolescents point to the importance of early intervention and prevention efforts targeting this group (McGorry & Mei, 2018).

Emotional symptoms were linked with both being bothered by and bothering siblings over time on the within‐person level, suggesting that emotional difficulties may contribute to experiencing more problems with siblings. Previous studies have similarly identified associations between mental health development in children and adolescents and parental mental distress (Speyer, Hall, et al., 2022) and harsh parenting (Hipwell et al., 2008; Lansford et al., 2011; Speyer, Hang, et al., 2022; Wang & Kenny, 2014). We extend these findings relating mental health with parental factors by providing evidence of temporal within‐person relationships between emotional difficulties and sibling relationship problems. We also observed strong concurrent within‐person associations between mental health problems and family environment factors. Specifically, conduct problems were primarily linked to externalised behaviour and disruptive interactions in the family (e.g., bothering siblings, fighting with parents), while emotional symptoms were related to negative internalised experiences (i.e., feeling negatively about one's family, being bothered by siblings).

Reflecting its substantial relationships with other mental health domains (i.e., hyperactivity/inattention) and sibling problems over time, emotional symptoms had the strongest outwards influence in the temporal network. It has been shown that affect states of different family members are associated in daily life, for example, if adolescents report irritability, it is more likely that their mothers report irritability at the next moment (Veenman et al., 2024). We find that emotional problems also predict negative family dynamics. A potential implication is that lessening of internalising difficulties may positively impact perceived aspects of the family environment.

Our results also unravel family dynamics across early adolescence, beyond their associations with mental health difficulties. We identified a bidirectional loop between lack of support and negative feelings towards family, suggesting that these mutually reinforce each other over time. More fighting with parents predicted more negative feelings towards family and less talking to parents. Although here is limited evidence pertaining to the specific mechanisms which underly the effectiveness of parenting programmes for child outcomes like mental health (Sanders et al., 2014), our findings suggest that the improvement of one aspect (e.g., adolescent‐perceived parental support) may also have a positive effect on other family dynamics (e.g., feeling more positively about one's family). As was the case for mental health difficulties, family environment factors exhibited stability across time. In the temporal network, negative feelings towards one's family and hyperactivity/inattention were strongly predicted by the included variables, while emotional symptoms, fighting with parents, and lacking family support had strong outward influence (i.e., were predictive of other variables).

### Limitations and future directions

Our findings should be interpreted in the context of several important limitations. First, the panelGVAR model only captures linear dynamics and lag‐1 relationships (i.e., only consecutive measurements are modelled). As such, nonlinearity or mechanisms operating at other timescales may not be captured by the model (Ebrahimi et al., 2021; Haslbeck & Ryan, 2022). For instance, the average time lag in this study was 2 years. Temporal relationships between mental health difficulties and the family environment also occur across shorter time‐intervals (e.g., in daily life; Veenman et al., 2024). Several edges were estimated to zero in the temporal network. It is plausible that different patterns of associations could have been observed if the interval between measurements was shorter (e.g., months). Previous studies have shown that interactions between adolescent mental health and family factors may differ across timescales (Boele et al., 2023; Bülow et al., 2025). Future studies could assess how adolescent mental health is related to family dynamics across timescales. Second, while we included multiple adolescent‐perceived family environment factors, this does not guarantee that all relevant family aspects were included. Third, our sample was recruited from the general population, and the extent to which our findings are generalisable to clinical populations is therefore unclear. Future studies could examine relationships between mental health and the family environment in clinical adolescent samples. Fourth, we used several composite scores in the estimated model, which do not account for measurement error. Fifth, we cannot exclude the potential impact of common method bias, as both mental health and family environment factors were measured using self‐report methods (Podsakoff et al., 2003). Future studies could integrate information across informants for example, including both self‐reported and parent‐reported measures, and evaluate if observed associations remain consistent. Sixth, several family factors were assessed using a small number of items (talking and quarrelling with parents) or a single item (family support), which may reduce reliability and validity. Finally, adolescence is a period of great change across developmental domains (Blakemore, 2019). A useful aim for future studies would be to examine potential age‐specificity in associations between adolescent mental health and the family environment.

## CONCLUSION

Our findings reveal the dynamic interplay between mental health difficulties and family environment factors in early adolescence. At the within‐person level, emotional symptoms predicted hyperactivity/inattention and problems with siblings at the next timepoint, while all mental health domains were strongly interrelated contemporaneously and exhibited considerable stability. We also uncovered family dynamics over time, including a bidirectional loop between lack of support and negative feelings towards family. Our results underscore the interrelatedness of mental health difficulties and the family environment, as well as temporal effects within and across symptom domains and family dynamics. Alleviating internalising problems in affected adolescents may help mitigate the development of other mental health difficulties and negative sibling dynamics over time.

## AUTHOR CONTRIBUTIONS

**Ludvig Daae Bjørndal**: Conceptualization; data curation; formal analysis; investigation; methodology; project administration; writing—original draft. **Omid V. Ebrahimi**: Supervision; writing—review and editing. **Sarah Bauermeister**: Supervision; writing—review and editing.

## CONFLICT OF INTEREST STATEMENT

The authors declare no conflicts of interest.

## ETHICAL CONSIDERATIONS

The University of Essex Ethics Committee has approved all data collection on Understanding Society main study, COVID‐19 surveys and innovation panel waves, including asking consent for all data linkages except to health records. Requesting consent for health record linkage was approved at Wave 1 by the National Research Ethics Service (NRES) Oxfordshire REC A (08/H0604/124), at BHPS Wave 18 by the NRES Royal Free Hospital & Medical School (08/H0720/60) and at Wave 4 by NRES Southampton REC A (11/SC/0274). Approval for asking consent for health record linkage and for the collection of blood and subsequent serology testing in the March 2021 wave of the COVID‐19 study was obtained from London—City & East Research Ethics Committee (21/HRA/0644). Approval for the collection of biosocial data by trained nurses in Waves 2 and 3 of the main survey was obtained from the National Research Ethics Service (Understanding Society—UK Household Longitudinal Study: A Biosocial Component, Oxfordshire A REC, Reference: 10/H0604/2). The biosocial data collection at IP12 ‘Understanding Society Health Innovation Panel: Biomeasure and health data collection from the Innovation Panel of the UK Household Longitudinal Study’ was approved by East of England—Essex Research Ethics Committee, Ref 19/EE/0146.

For further details on the various committees which have provided ethical approval of the study and its components, see: https://www.understandingsociety.ac.uk/documentation/mainstage/user‐guides/main‐survey‐user‐guide/ethics/.

## Supporting information

Supporting Information S1

## Data Availability

The data used in this paper are available to researchers by applying to the UK Data Service (https://ukdataservice.ac.uk).
