# Supplementary material for: The dynamic interplay between mental health difficulties and the family environment in early adolescence
Source: JCPP Adv. 2025 Aug 12;6(2):e70037. doi: 10.1002/jcv2.70037 (PMC13260696; doi:10.1002/jcv2.70037)
Supplement: Supplementary file 1 — Supporting Information S1 [file JCV2-6-e70037-s001.docx]

**Supplementary Materials**

For manuscript entitled: “The dynamic interplay between the family environment and mental health difficulties in early adolescence”

Date: 22.03.2025

Ludvig Daae Bjørndal, PhD (Corresponding author),

University of Oslo

E-mail: [l.d.bjorndal@psykologi.uio.no](mailto:l.d.bjorndal@psykologi.uio.no)

**Table of Contents**

Table S1. Items Included in Analyses.

Table S2. Descriptive Statistics in the Analytic Sample.

Table S3. Correlation Between Variables Across Waves of Data Collection.

Figure S1. Sample Selection Process in the UK Understanding Society Survey.

Figure S2. Strength Centrality Contemporaneous Network.

**Table S1.**

*Variables Included in the Analyses.*

| **Variable** | **Item** | **Response format** | **Item / sum score in model** |
| --- | --- | --- | --- |
| Emotional symptoms | I get a lot of headaches, stomach-aches or sickness | Not true / Somewhat true / Certainly true | Sum score |
|  | I worry a lot |  | Sum score |
|  | I am often unhappy, down-hearted or tearful |  | Sum score |
|  | I am nervous in new situations. I easily lose confidence |  | Sum score |
|  | I have many fears, I am easily scared |  | Sum score |
| Hyperactivity | I am restless, I cannot stay still for long | Not true / Somewhat true / Certainly true | Sum score |
|  | I am constantly fidgeting or squirming |  |  |
|  | I am easily distracted, I find it difficult to concentrate |  |  |
|  | I think before I do things |  |  |
|  | I finish the work I'm doing |  |  |
| Conduct problems | I get very angry and often lose my temper | Not true / Somewhat true / Certainly true | Sum score |
|  | I usually do as I am told |  |  |
|  | I fight a lot. I can make other people do what I want |  |  |
|  | I am often accused of lying or cheating |  |  |
|  | I take things that are not mine from home, school or elswhere |  |  |
| Family support | Do you feel supported by your family, that is the people who live with you? | In most or all of the things I do / In some of the things I do / I do not feel supported by my family | Item |
| Feel about family | [Please tick the box which comes closest to expressing how you feel about (...)] Your family? | Completely happy (1) - Not at all happy (7) | Item |
| Being bothered by siblings | How often do any of your brothers or sisters do any of the following to you at home? | |  |
|  | Hit, kick or push you | Never / Not much (1-3 times in the last 6 months) / Quite a lot (more than 4 times in the last 6 months) / A lot (a few times every week | Sum score |
|  | Take your belongings |  |  |
|  | Call you nasty names |  |  |
|  | Make fun of you |  |  |
| I bother siblings | How often do you do any of the following to your brothers or sisters at home? |  |  |
|  | Hit, kick, or push them | Never / Not much (1-3 times in the last 6 months) / Quite a lot (more than 4 times in the last 6 months) / A lot (a few times every week) | Sum score |
|  | Take their belongings |  |  |
|  | Call them nasty names |  |  |
|  | Make fun of them |  |  |
| Talk to parents | How often do you talk to your mother, about things that matter to you? | Most days / More than once a week / Less than once a week / Hardly ever | Sum score |
|  | How often do you talk to your father, about things that matter to you? |  |  |
| Fight with parents | Most children have occasional quarrels with their parents. | |  |
|  | How often do you quarrel with your mother? | Most days / More than once a week / Less than once a week / Hardly ever | Sum score |
|  | How often do you quarrel with your father? |  |  |

**Table S2.**

*Descriptive Statistics in the Analytic Sample*.

| **Variable** | **W1** | **W2** | **W3** |
| --- | --- | --- | --- |
|  | **Mean (SD)** | | |
| Emotional symptoms | 2.83 (2.17) | 2.86 (2.31) | 3.31 (2.48) |
| Conduct problems | 2.15 (1.82) | 2.03 (1.79) | 2.00 (1.78) |
| Hyperactivity/inattention | 3.91 (2.32) | 3.85 (2.39) | 4.00 (2.39) |
| Lacking family support | 1.17 (0.41) | 1.20 (0.44) | 1.28 (0.49) |
| Negative feelings towards family | 1.35 (0.79) | 1.63 (1.04) | 1.96 (1.23) |
| Fighting with parents | 3.39 (1.80) | 3.36 (1.65) | 3.38 (1.62) |
| Talking to parents | 5.35 (2.02) | 4.97 (2.03) | 4.70 (2.03) |
| Bothered by sibling | 7.70 (3.07) | 7.58 (3.10) | 7.24 (2.91) |
| Bothering my siblings | 6.77 (2.63) | 6.80 (2.65) | 6.69 (2.54) |

**Table S3.**

*Correlation Between Variables Across Waves of Data Collection*.

|  | **Correlation (*r*) across waves (W)** | | |
| --- | --- | --- | --- |
| **Variable** | W1 and W2 | W1 and W3 | W2 and W3 |
| Emotional symptoms | .44 | .34 | .57 |
| Conduct problems | .51 | .44 | .56 |
| Hyperactivity/inattention | .52 | .41 | .57 |
| Lacking family support | .20 | .16 | .38 |
| Negative feelings towards family | .28 | .30 | .46 |
| Fighting with parents | .34 | .27 | .47 |
| Talking to parents | .33 | .25 | .47 |
| Bothered by sibling | .43 | .33 | .50 |
| Bothering my siblings | .42 | .39 | .53 |

**Figure S1.**

*Sample Selection Process in the UK Understanding Society Survey.*

**Figure S2.**

*Strength Centrality Contemporaneous Network.*


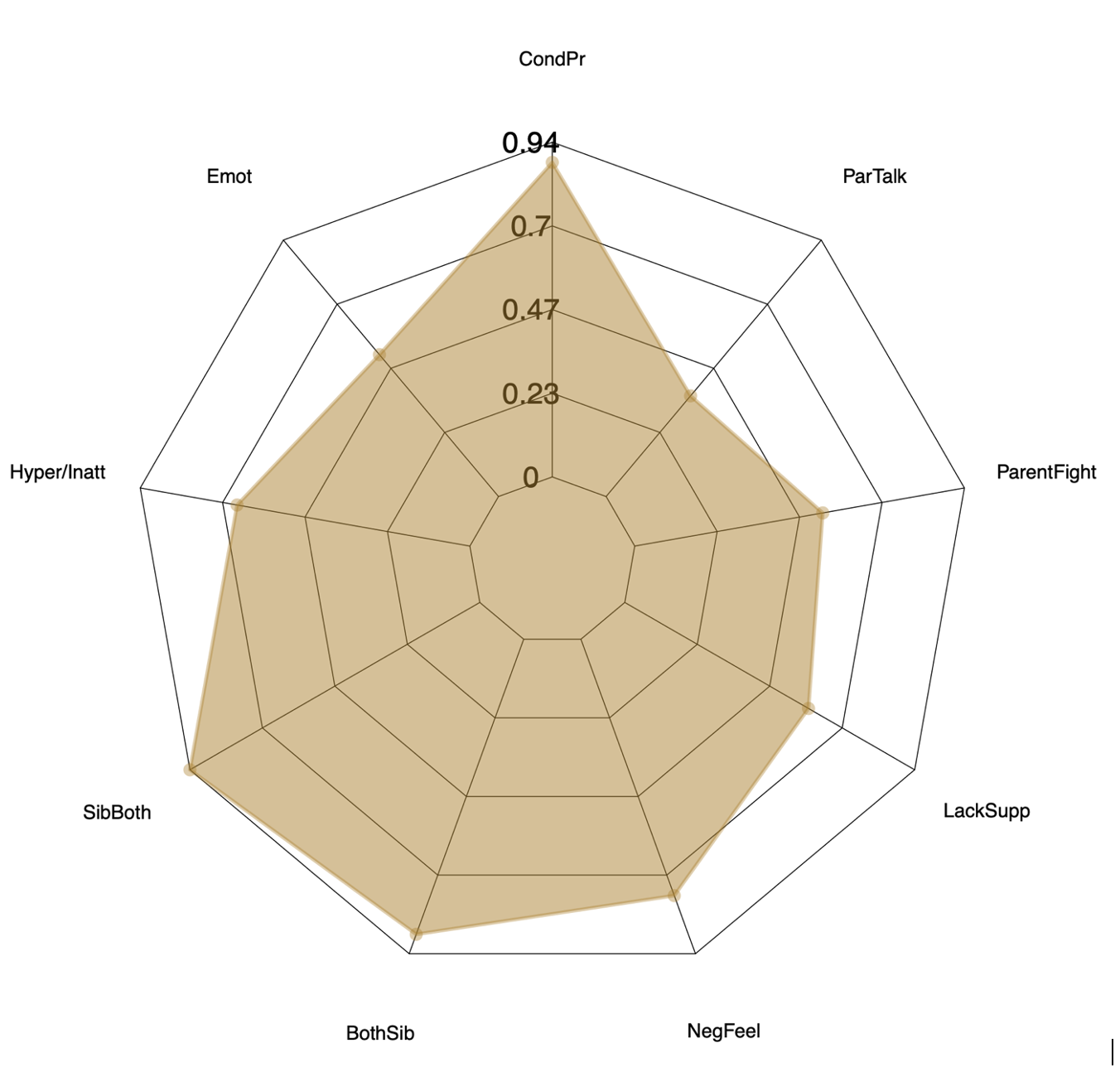


*Note*. SibBoth: Sibling bother me. ParTalk: Talking to parents about things that matter to me. NegFeel: Feeling negative towards family. ParentFight: Fights or quarrels with parents. LackSupp: Lacking support from family. BothSib: I bother my siblings. Hyper/Inatt: Hyperactivity/inattention.
